# Automatic High-Resolution Operational Modal Identification of Thin-Walled Structures Supported by High-Frequency Optical Dynamic Measurements

**DOI:** 10.3390/ma17204999

**Published:** 2024-10-12

**Authors:** Tongfa Deng, Yuexin Wang, Jinwen Huang, Maosen Cao, Dragoslav Sumarac

**Affiliations:** 1 School of Civil and Surveying & Mapping Engineering, Jiangxi University of Science and Technology, Ganzhou 341000, China; 2 Jiangxi Province Key Laboratory of Environmental Geotechnical Engineering and Hazards Control, Jiangxi University of Science and Technology, Ganzhou 341000, China; 3 College of Mechanics and Engineering Science, Hohai University, Nanjing 211100, China; 4 Department of Technical Sciences, Civil Engineering, State University of Novi Pazar, 36300 Novi Pazar, Serbia

**Keywords:** thin-walled structure, optical dynamic measurement, automated operational modal identification, clustering algorithm

## Abstract

High-frequency optical dynamic measurement can realize multiple measurement points covering the whole surface of the thin-walled structure, which is very useful for obtaining high-resolution spatial information for damage localization. However, the noise and low calculation efficiency seriously hinder its application to real-time, online structural health monitoring. To this end, this paper proposes a novel high-resolution frequency domain decomposition (HRFDD) modal identification method, combining an optical system with an accelerometer for measuring high-accuracy vibration response and introducing a clustering algorithm for automated identification to improve efficiency. The experiments on the cantilever aluminum plate were carried out to evaluate the effectiveness of the proposed approach. Natural frequency and damping ratios were obtained by the least-squares complex frequency domain (LSCF) method to process the acceleration responses; the high-resolution mode shapes were acquired by the singular value decomposition (SVD) processing of global displacement data collected by high-speed cameras. Finally, the complete set of the first nine order modal parameters for the plate within the frequency range of 0 to 500 Hz has been determined, which is closely consistent with the results obtained from both experimental modal analysis and finite element analysis; the modal parameters could be automatically picked up by the DBSCAN algorithm. It provides an effective method for applying optical dynamic technology to real-time, online structural health monitoring, especially for obtaining high-resolution mode shapes.

## 1. Introduction

Thin-walled structures are widely used in engineering, such as the aeronautical part and thin-walled concrete structures, because of their advantages of being lightweight and their high load-bearing capacity. The aeronautical part is prone to chatter in high-speed milling due to the low stiffness [1]. For the thin-walled concrete structures, researchers pay more attention to the sustainability discussions of existing building structures [2,3]. Therefore, a condition assessment of thin-walled structures is essential in deciding whether to strengthen existing structures or identify damage such as chatter [1,4]. There are many studies on the vibration-based testing of structures [5], and modal analysis is the most common dynamin test.

It is difficult to obtain reliable input excitation data, so operating modal analysis (OMA) [6,7,8,9] has been developed based on the vibration response data of the output only. Currently, the most common method for collecting vibration response data is the contact measurements technique [10], which involves attaching acceleration sensors, strain gauges, or using displacement meters. In real-case scenarios, structures can be instrumented only with a limited number of sensors [11]. Especially for thin-walled structures, their thickness is much smaller than the other two dimensions. A lot of contact sensors attached will have a significant effect on their dynamic characteristics. Using a limited number of accelerometers, researchers have developed modal expansion for damage detection with incomplete mode shapes [12]. In addition to modal expansion, considering the root defects of contact measurement technology, non-contact measurement technology has been developed, such as acoustic holography [13], laser profilometer [14], optical flow imaging [15], and digital image correlation measurement (DIC) [16].

Modern optical mechanics has become a powerful tool for researchers because of its excellent characteristics of high accuracy, full-field measurement, and non-contact. The rapid development of digital image processing hardware and software has brought new development opportunities to optical measurement mechanics, and digital image correlation (DIC) technology [17] has emerged. This method sprays speckles on the surface of the object being measured, focuses on the speckle field before and after deformation, and obtains the strain and displacement information of the structure. For thin-walled structures, this paper proposed a high-resolution modal identification method by using high-frequency optical dynamic measurement.

Due to the limited number of the typical tools for optical dynamic measurement technology applied to obtain modal parameters, there are many studies on its application for modal analysis. Ha et al. [18] used an optical system to measure the vibration of an artificial wing for evaluating its performance. The spectrum averaging and filtering were used in this study to reduce the noise. The results show that non-contact measurement is a potentially promising method for the modal analysis of lightweight and miniature structures. Poozesh et al. [19] constructed a multi-camera measurement system based on dynamic spatial data stitching technology. The results show that the optical method is expected to become a powerful tool for identifying the dynamic characteristics of large-area engineering structures. Molina-Viedma et al. [20] proposed an experimental modal analysis method and they pointed out that the optical measurement system has no additional mass and will not affect the dynamic response of the tested structure. As long as the measured displacement response is analyzed, the natural frequency and operating deflection shape (ODS) of the structure can be obtained. Kumar et al. [21] developed a real-time, high-speed, non-contact method that is suitable for identifying the dynamic parameters of light and flexible structures. Cuadrado et al. [22] conducted a study using 3D-DIC for model updating and found that although fewer natural frequencies were obtained, the identified mode shapes were smoother. Frankovský et al. [23] pointed out that using the camera can capture the full-field response at the same time and under the same excitation conditions compared with other non-contact measurement methods. All of the above studies show that optical dynamic measurement has prominent features in obtaining high-resolution modal parameters.

Furthermore, to realize the real-time and online evaluation of the structural operation state, it is necessary to develop an automatic identification of modal parameters. The stability diagram [24], serving as an effective modal indication tool, has been used to identify the true modes for a long time. The process of automatically identifying true modes from stability diagrams can be transformed into the process of classifying stable points with similar characteristics, and fuzzy clustering algorithms happen to be able to achieve an intelligent classification of data points [25]. According to different clustering algorithms, the current automatic modal parameter identification methods can be divided into the following three categories [26]: automatic modal parameter identification based on the hierarchical clustering algorithm, non-hierarchical clustering algorithm, and density clustering algorithm. The density-based clustering algorithm stands out for its scalability and exhibits less sensitivity to input threshold or parameters. Crucially, its ability to filter out irregular abnormal data points (noise) renders it highly appropriate for the automated identification of modal parameters in choosing accurate poles.

The typical research on automatic operational modal analysis (AOMA) using density clustering is outlined below. Considering the parameter setting problem, Civera et al. [27] proposed an algorithm applied to a real engineering case. It is fully automated, including the data-driven setting of the input parameters of the algorithm. Boroschek et al. [28] developed an automatic method with three stages for interpreting stability diagrams. He et al. [29] developed a framework for the AOMA of bridges through the integration of density-based clustering theory and the uncertainty of estimated frequencies. Ye [30] et al. proposed an AOMA algorithm based on DBSCAN clustering and applied it to the benchmark model. The study pointed out that the clustering algorithm generally has subjective input parameter selection, complex algorithm theory, and human intervention.

In summary, the use of optical dynamic measurement for OMA is very widespread, but there are still some shortcomings: ① Most of the modal analysis theories or methods proposed do not discuss the influence of calculation time. Obtaining high-resolution mode shapes from displacement data is time-consuming. The existing proposed methods may not be suitable for real-time monitoring and the online evaluation of structures if the calculation cost is too high. ② Noise is unavoidable in the actual measurement process. Due to environmental noise, the ability of the camera to precisely detect modes is limited. The recognition capability of high-order modes is subpar, potentially being entirely overwhelmed by noise. The noise robustness of the optical method needs to be improved. ③ There is much research on AOMA, but most of it is based on the traditional contact measurement technique, which may not be suitable for optical measurement. It is necessary to develop AOMA technology suitable for optical dynamic measurement.

The further development and application of optical dynamic measurement in the field of modal analysis and structural health monitoring will be limited because of these shortcomings. In this paper, an OMA method based on the measurement technique by combining high-speed cameras and one accelerometer, termed high-resolution frequency domain decomposition (HRFDD), is developed. It has high accuracy, high efficiency, and no order missing of modal parameters with a suitable identification algorithm. Furthermore, the modal distance is defined based on natural frequency and mode shape to develop AOMA through the DBSCAN algorithm.

The remainder of this paper is organized as follows. In Section 2, the theoretical background is introduced and HRFDD is proposed for the high-resolution OMA of thin-walled structures. In Section 3, a clustering algorithm is proposed to realize the AOMA, and a numerical study on a ten-story-type frame structure is conducted to validate the accuracy and performance of the proposed algorithm. In Section 4, taking a cantilever rectangular aluminum plate as an example, the modal experiment of the thin-walled structure is carried out to verify the capability of the proposed, improved OMA approach, HRFDD, and the AOMA algorithm. Finally, the conclusions are summarized in Section 5.

## 2. Noise-Robust High-Resolution Modal Identification Method

### 2.1. Classical Frequency Domain Operational Modal Analysis

#### 2.1.1. Peak Picking Method (PP)

In the field of structural modal identification, the PP method is the most commonly used OMA method [31]. For systems with relatively small damping and low coupling between adjacent modes, the PP method has higher identification accuracy. According to the theory of random vibration, the PSD matrix $R_{\mathrm{xx}}$ of the input excitation and the PSD matrix $G_{\mathrm{yy}}$ of the output response satisfy the following relationship [7]:

(1) $$G_{\mathrm{yy}}\left(\omega \right)=H\left(\omega \right)R_{\mathrm{xx}}H{\left(\omega \right)}^{H}$$

where $G_{\mathrm{yy}}\in \;C^{ο\times ο}$, $R_{\mathrm{xx}}\in \;C^{i\times i}$, ο is the number of outputs, i is the number of inputs, $H\left(\omega \right)$ is the frequency response function, and ${\left[•\right]}^{H}$ represents the Hermitian transpose operation. Under white noise excitation (uniform excitation of the spectrum), when ω approaches the r-th natural frequency $\omega_{r}$, $G_{\mathrm{yy}}\left(\omega \right)$ can be expressed as follows:

(2) $$G_{\mathrm{yy}}\left(\omega_{r}\right)\approx \beta_{r}\cdot \varphi_{r}\varphi_{r}^{H}$$

where $\beta_{r}$ is a constant associated with the matrix $R_{\mathrm{xx}}$ and the r-th modal parameter, which includes the natural frequency and damping ratio, and $\varphi_{r}$ is the *r*-th mode shape. Equation (2) shows that each column in the $G_{\mathrm{yy}}\left(\omega_{r}\right)$ matrix is the ratio of the *r*-th order shape. For this reason, the mode shape can be obtained by normalizing the column vector of $G_{\mathrm{yy}}\left(\omega_{r}\right)$, and the natural frequency can be picked up from the peak of the PSD amplitude curve of the output response. For the damping ratio, the half-power bandwidth method can be used to obtain the following:

(3) $$\xi_{r}=\frac{\omega_{b}-\omega_{a}}{2\omega_{r}}$$

where $\xi_{r}$ represents the *r*-th damping ratio of the system, and $\omega_{a}$ and $\omega_{b}$ are the frequencies corresponding to the half-power point in the PSD amplitude curve of the output response, respectively.

#### 2.1.2. Enhanced Frequency Domain Decomposition Method (EFDD)

It is difficult to identify close-spaced modes by the PP method, while the EFDD method uses singular value decomposition (SVD) to decouple the multi-degree-of-freedom PSD functions into a series of single-degree-of-freedom PSD functions, which allows EFDD to have advantages in identifying close-spaced modes. Under white noise excitation, when ω approaches the *r*-th natural frequency $\omega_{r}$, $G_{\mathrm{yy}}\left(\omega_{r}\right)$ can be written as the following equation [32,33]:

(4) Gyyωr≈φr*diag2Reαriω−λrφrT=φr*diag2αrξrωrξrωr2+ω−ωdr2φrT

where $\alpha_{r}$ is a real number, $\lambda_{r}$ is the *r*-th pole of the system, $\omega_{dr}$ is the natural frequency with damping, and Equation (4) above is actually the SVD form of matrix $G_{\mathrm{yy}}\left(\omega_{r}\right)$. The EFDD method first performs SVD decomposition on the matrix $G_{\mathrm{yy}}\left(\omega_{r}\right)$ of all frequency points ω, and identifies natural frequencies according to the peak values of the singular value spectrum, and the first-order singular vector of peak points is the mode shape. For the damping ratio, inverse Fourier transform may be performed on the singular value spectrum to obtain the autocorrelation function, and linear fitting may be performed on the extreme points of the autocorrelation function in logarithmic coordinates to obtain the logarithmic decrement δ, expressed as the following:

(5) $$\delta =\frac{2}{k}ln\left(\frac{R_{1}}{R_{k}}\right)$$

where $R_{1}$ is the first peak point of the autocorrelation function and $R_{k}$ is the k-th extreme point of the autocorrelation function. When it is obtained, the damping ratio can be calculated with the following:

(6) $$\xi =\frac{\delta }{\sqrt{\delta^{2}+4\pi^{2}}}$$

#### 2.1.3. Least-Squares Complex Frequency Domain Method (LSCF)

The LSCF method is a fast and accurate method for modal analysis. Under white noise excitation, the FRF of the structure can be approximated by a PSD function. Therefore, the PSD function can be fitted into a right matrix-fraction model (RMFM) by using the LSCF method, and RMFM is expressed as the following equation [34]:

(7) $$\left[G(\omega )\right]=\left[B\left(\omega \right)\right]{\left[A\left(\omega \right)\right]}^{-1}$$

where $G\left(\omega \right)\in C^{l\times t}$ is the theoretical power spectral density matrix, *l* is the number of outputs, *t* is the number of reference outputs, $B\left(\omega \right)\in C^{l\times t}$ and $A\left(\omega \right)\in C^{t\times t}$ are matrix polynomials, namely, the numerator and denominator of RMFM, respectively. $G_{h}(\omega )$ is the *h*-th row of RMFM and can be expressed as the following:

(8) $$\left[G_{h}\left(\omega \right)\right]=\left[B_{h}\left(\omega \right)\right]{\left[A_{h}\left(\omega \right)\right]}^{-1}=\sum_{n=0}^{p}\Omega_{n}\left(\omega \right)\langle \beta_{hn}\rangle \times {\left(\sum_{n=0}^{p}\Omega_{n}\left(\omega \right)\left[\alpha_{n}\right]\right)}^{-1},h=1,2,3,\ldots ,l$$

where $\Omega_{n}\left(\omega \right)=e^{j\omega \cdot \Delta t\cdot p}$ is the basis function, *j* is the imaginary unit, Δt is the sampling interval time, and *p* is the order of RMFM. $\beta_{hn}\in C^{l\times t}$ and $\alpha_{n}\in C^{t\times t}$ are the coefficients of RMFM to be solved.

It is a non-linear problem to fit the measured PSD estimation matrix directly with RMFM. Therefore, the non-linear fitting is converted into linear fitting by the following formula:

(9) $$\varepsilon_{h}=w_{h}\left(\omega \right)\left(B_{h}\left(\omega \right)-{\hat{G}}_{h}\left(\omega \right)A\left(\omega \right)\right)$$

where $\varepsilon_{h}$ is the fitting residual, $w_{h}\left(\omega \right)$ is the weight function, and ${\hat{G}}_{h}\left(\omega \right)$ is the *h*-th row of the measured PSD matrix. After linearization, linear least-squares fitting is performed on Equation (9) to calculate the coefficients $\alpha_{n}$ and $\beta_{hn}$. The companion matrix can be constructed by using the coefficient $\alpha_{n}$. The pole λ of the system can be obtained by performing eigenvalue decomposition on the companion matrix, and the natural frequency and damping ratio can be obtained from the poles of the system. For mode shapes, calculations are performed using the least-squares frequency-domain method [35]:

(10) $$\left[G\left(\omega \right)\right]=\sum_{r=1}^{m}\left(\frac{\left\{\varphi_{r}\right\}l_{r}^{T}}{j\omega -\lambda_{r}}+\frac{\left\{\varphi_{r}^{*}\right\}l_{r}^{H}}{j\omega -\lambda_{r}^{*}}\right)+\frac{\left[LR\right]}{j\omega }+j\omega \left[UR\right]$$

where $\varphi_{r}$ is the *r*-th mode shape, $l_{r}$ is the *r*-th operating reference factor vector, and LR and UR are the low-frequency residual term and the high-frequency residual term, respectively.

### 2.2. High-Resolution Frequency Domain Decomposition Method (HRFDD)

This paper puts forward an improved method (HRFDD) for modal parameter identification by weighing the advantages and disadvantages of contact and non-contact vibration measurement techniques. In the process of modal identification, the method balances the precision of the identified modal parameter and calculation efficiency. It realizes the application of the stereo optical dynamic measurement system to modal parameter identification, especially realizing that the natural frequency, damping ratio, and mode shape of the thin-walled structures can all be obtained with high-resolution. Here, high-resolution for mode shape means high spatial resolution and a high accuracy of the natural frequency and damping ratio. The method comprehensively considers the optimization of OMA from three aspects.

**Figure 1:**
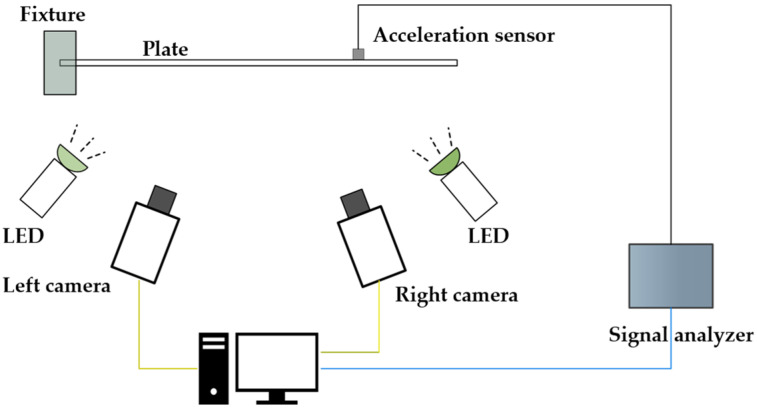
High-frequency optical dynamic measurement system for high-resolution modal identification.

Firstly, it is difficult to obtain an accurate natural frequency and damping ratio without missing modes when optical dynamic measurement is used to obtain high-resolution modal shape, due to noise and the limitation of camera memory, while the acceleration sensor can obtain a high precision natural frequency and damping ratio. In this paper, a measurement method combining an acceleration sensor and high-speed camera is proposed, which adds a high-precision acceleration sensor to assist the response measurement. The proposed dynamic response measurement system is as Figure 1 illustrates. In this measurement system, high-speed cameras are adopted to collect the full-filed displacement of the structure, and one acceleration is attached for collecting higher accuracy response data than cameras. Through the combination of two kinds of measurement technologies, the inaccuracy of optical dynamic measurement caused by noise in obtaining high-resolution modal parameters is solved.

Secondly, according to the different characteristics of response data measured by the acceleration sensor and camera, different methods are adopted to process the data. The LSCF method is used to obtain the natural frequency and damping ratio from acceleration data because of the high identification accuracy. The SVD method with the noise reduction effect is used to acquire mode shapes from preprocessed full-filed optical displacement data with a low signal-to-noise ratio.

Finally, considering the problem of computational efficiency in dealing with displacement data from optical measurement, the Welch power spectral density estimation method is introduced to calculate the PSD matrix directly at natural frequency. The PSD functions where other discrete frequency points are not calculated, which greatly reduces the calculation amount without losing the identification accuracy. It is worth noting that when the EFDD method is used to identify modal parameters, the function *cpsd* will be called to calculate the PSD function in MATLAB. It will calculate the PSD function values of all discrete points, and users cannot control the *cpsd* function to only calculate and output the PSD function values of interest frequency points.

The Welch method initially segments the response sequence, applies windowing to these segments, and subsequently calculates the average after transforming the data from the time domain to the frequency domain. It is used to calculate the cross-power spectral density ${\hat{G}}_{12}\left(\omega \right)$ of response signals $x_{1}\left(t\right)$ and $x_{2}\left(t\right)$, and the calculation of the Equation [36] is as follows:

(11) $${\hat{G}}_{12}\left(\omega \right)=\frac{1}{N_{b}}\sum_{k=1}^{N_{b}}\left\{\frac{\sum_{t=1}^{M}w\left(t\right)x_{1}\left(t\right)e^{-j\omega t}}{MP}\cdot conj\left[\sum_{t=1}^{M}w\left(t\right)x_{2}\left(t\right)e^{-j\omega t}\right]\right\}$$

where ω is the circular frequency, $N_{b}$ is the total number of segments dividing the response signal, *k* represents the *k*-th segment signal, *M* is the length of each segment of data, $w\left(t\right)$ is a non-rectangular window function applied to reduce energy leakage, $conj\left(•\right)$ denotes a conjugate operation, and *P* denotes a normalization factor, which can be expressed as follows:

(12) $$P=\frac{1}{M}\sum_{t=1}^{M}{\left|w\left(t\right)\right|}^{2}$$

When $x_{1}\left(t\right)=x_{2}\left(t\right)$, ${\hat{G}}_{12}\left(\omega \right)$ is the self-power spectral density, when $x_{1}\left(t\right)\neq x_{2}\left(t\right)$, ${\hat{G}}_{12}\left(\omega \right)$ is the cross-power spectral density. Due to the implementation of averaging, the PSD estimated by the Welch method exhibits enhanced noise robustness.

The proposed method in this paper uses Equation (11) to calculate only the PSD function value $G_{1,2}\left(\omega_{i}\right)$ at the natural frequency $\omega_{i}$, so it does not include redundant calculations.

**Figure 2:**
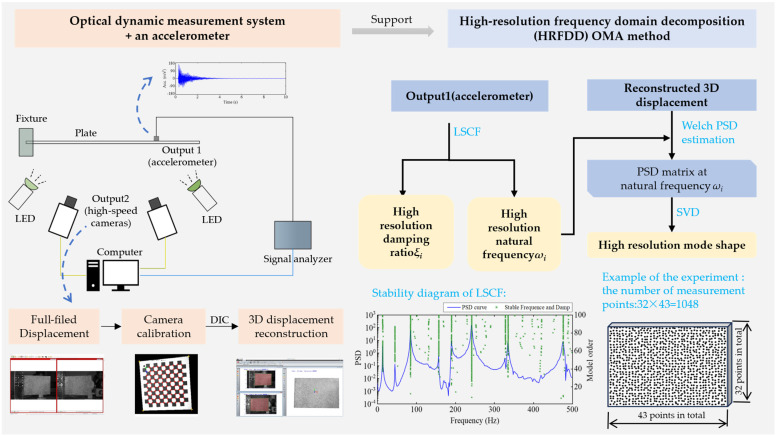
Flowchart of the improved operating modal analysis approach.

The flowchart of the proposed OMA method HRFDD is shown in Figure 2. In order to further improve the identification efficiency, this paper proposes a DBSCAN-based AOMA algorithm applied to the LSCF stability diagram. The procedure of AOMA is to add clustering to the LSCF stability diagram on the basis of the OMA method.

## 3. The Automatic Identification Method Based on the Clustering Algorithm

In practical engineering applications, the stable points in the LSCF stability diagram are not arranged strictly according to the rules due to the large amount of data and the interference of uncertain factors such as noise. The selection of true poles usually requires manual intervention and is time-consuming. The problem of automatically picking true poles can be transformed into a classification problem of stable points. Therefore, this paper proposes an automatic true pole selection method based on the DBSCAN density clustering algorithm in the field of machine learning and carries out numerical simulations and experiments to verify the effectiveness of this method.

### 3.1. DBSCAN Algorithm

DBSCAN is the abbreviation for density-based spatial clustering of application with noise. It is a typical density-based clustering method. The main idea of the DBSCAN method is to find all the dense regions of sample points from the core point and cluster these dense regions into different clusters. This method has two input parameters: neighborhood radius Eps and minimum number MinPts. For a detailed introduction to using DBSCAN and its clustering procedure, see Ester et al. [37].

**Figure 3:**
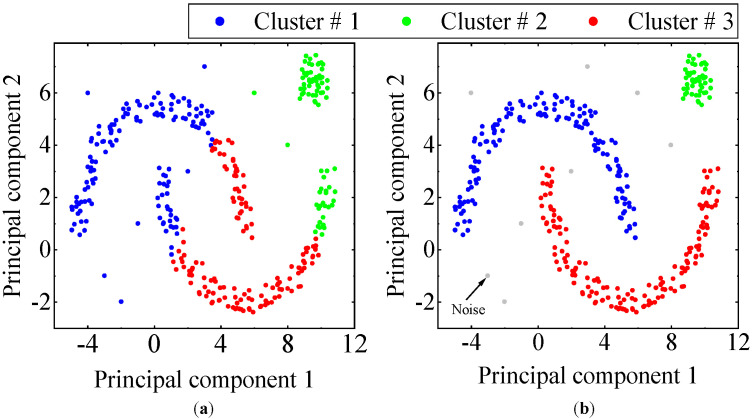
The results of clustering: (**a**) K-means and (**b**) DBSCAN.

It is noteworthy that DBSCAN stands out from the conventional K-means clustering method as it can identify and remove outliers (noise points) without requiring prior knowledge of the number of clusters. Figure 3 illustrates the clustering results achieved by K-means and DBSCAN on the identical dataset. As can be seen from the figure, DBSCAN is robust against outliers and can accurately cluster clusters of arbitrary size and shape. For the automated identification of stability diagrams, DBSCAN can effectively eliminate the influence of false poles.

### 3.2. Automatic Selection of Stability Poles Based on Density Clustering Algorithm

#### 3.2.1. Modal Distance

$d\left(i,j\right)$ is defined by combining natural frequency and mode shape as a criterion for the classification of stable points by the clustering algorithm [38]. The smaller the modal distance, the more similar the stable points; conversely, the larger the modal distance, the greater the difference between the stable points.

(13) $$d\left(i,j\right)=c_{1}\cdot \left|\frac{f^{\left(i\right)}-f^{\left(j\right)}}{\max\left(f^{\left(i\right)},f^{\left(j\right)}\right)}\right|+c_{2}\cdot \left[1-\mathrm{MAC}\left(\varphi_{i},\varphi_{j}\right)\right]$$

where $d\left(i,j\right)$ represents the modal distance between the *i*-th and *j*-th stable points, and $f^{\left(i\right)}$ and $f^{\left(j\right)}$ are the natural frequencies of the *i*-th and *j*-th stable points, respectively. $\varphi_{i}$ and $\varphi_{j}$ are the mode shapes at the *i*-th and *j*-th stable points, respectively. The MAC is the modal assurance criterion [39,40]. The closer the MAC value is to 1, the higher the modal correlation is. The coefficient $c_{1}$ and $c_{2}$ are weighting coefficients for frequency and mode shape, respectively, which reflect the contribution of frequency and damping to the overall modal distance. In practical engineering, the identification accuracy of natural frequency is usually higher than that of mode shape, so $c_{1}$ should be set to a larger value than $c_{2}$. To find the optimal setting, allow one of the coefficients to start at 0 and increase to 1 with a gradient of 0.1. After a large number of tests, when $c_{1}=0.6$, $c_{2}=0.4$, the clustering effect is the best.

#### 3.2.2. Parameter Determination

For the automatic identification of true poles, *Eps* is equivalent to the accuracy of modal identification. When the modal distance $d\left(i,j\right)$ between two stable points is less than *Eps*, the two stable points are considered to belong to the same order mode. When constructing the stability diagram, the threshold standard of Equation (18) in Section 4.2 is adopted, that is, the frequency variation range of stable points is 3%, and the variation range of the damping ratio is 8%. Therefore, when DBSCAN is used to further classify these stable points, the frequency identification error must be less than 3%. Considering the accuracy of clustering results, the frequency and the mode shape identification accuracy are set to 0.01 in this paper [30]. Combined with Equation (13), *Eps* can be determined by the following equation:

(14) $$Eps=c_{1}\times 0.01+c_{2}\times 0.01=0.6\times 0.01+0.4\times 0.01=0.01$$

*MinPts* values are generally related to the model order *p* set in the LSCF method. By combining engineering practice with a large number of tests, *MinPts* values can be determined by Equation (15):

(15) $$MinPts=\left[\left(p_{\max}-p_{\min}+1\right)\times 10\%\right]$$

where [•] represents the rounding operation. It should be noted that in order to avoid missing modal orders, it is advisable to set smaller *MinPts* in practical applications, but it may cause the DBSCAN algorithm to cluster a small number of false poles caused by noise into a cluster, resulting in the loss of true modes. The number of stable points in false pole clusters is often lower than that of true pole clusters. Therefore, the three sigma criterion is introduced to eliminate the small number of clusters as outliers and eliminate the influence of the *MinPts* parameter setting.

#### 3.2.3. Clustering Procedure

The proposed automatic identification clustering process is divided into two steps. The first step is to remove the noise points that are not clustered after clustering, and the second step is to eliminate false pole clusters by using the three sigma criterion. The significance of choosing interval $\left(\mu -3\sigma ,\mu +3\sigma \right)$ is that according to normal distribution, the probability that the number of normal stable points are in this interval is 99.74%. Since the number of stable points of false pole clusters is minimal and usually not in the interval $\left(\mu -3\sigma ,\mu +3\sigma \right)$, the probability that the rejected clusters are false pole clusters is 99.74%. The last remaining clusters are regarded as true pole clusters, and the average value of each cluster is taken as the representative value of the modal parameters of this order. The specific process is as follows (Algorithms 1 and 2):
**Algorithm 1** Removing the noise points through DBSCAN algorithm**Input:** Dataset data_points (stable points) **D**, radius ***Eps***, minimum points ***MinPts*****Output:** Cluster labels for each data point 1 mark all data_points as “unvisited” and set the cluster index cluster_index = 0;  % Initialize all data_points2 for each point in data_points:  if point is unvisited:    mark point as visited    % Find neighbors of the point within radius ***Eps***    neighbors = find_neighbors(point, data_points, *Eps*)    % If the point is a core point (has at least MinPts neighbors)    if len(neighbors) ≥ ***MinPts***:      % Increment cluster index        cluster_index = cluster_index + 1        % Expand cluster from the core point        expand_cluster(point, neighbors, cluster_index) end for;3 set the cluster index to all points that were not assigned to a cluster cluster_index = −1;% label noise points

**Algorithm 2** Eliminating false poles based on the Three Sigma Criterion**Input:** The number of stable points in each cluster ***N_i_*****Output:** The clusters of true poles 1 The number of stable points in each cluster after clustering is regarded as a group of data to be inspected;2 Find the mean μ and standard deviation σ of the data to be examined;3 Construct the range fluctuation interval (μ−3σ, μ+3σ);4 Iterate over each cluster:   for each cluster    if the mean is out the interval (μ−3σ, μ+3σ)
    mark it as cluster of false pole    end if  end for5 Eliminate the cluster of false pole and find the clusters of true poles

### 3.3. Numerical Simulation Verification

**Figure 4:**
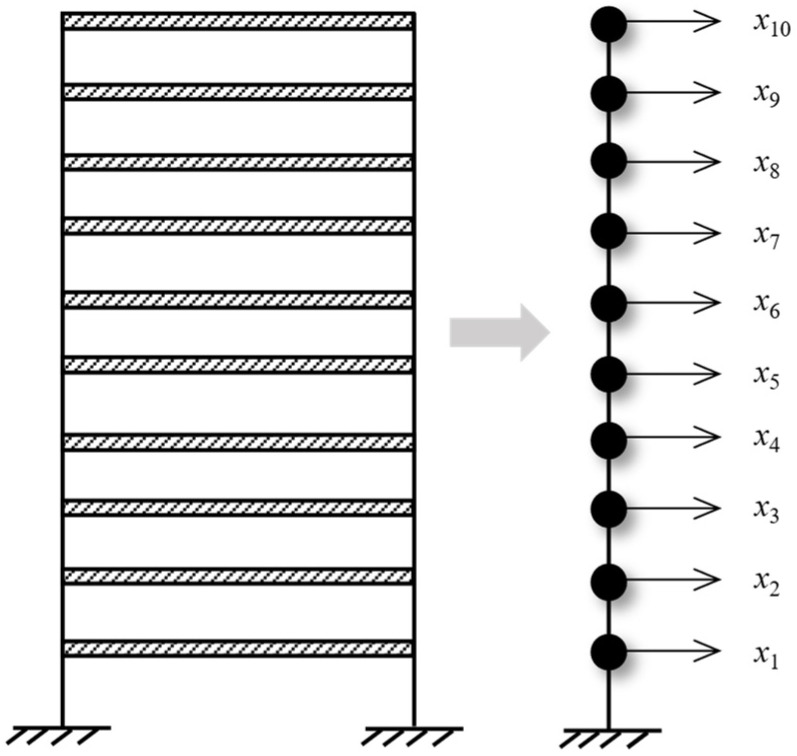
Equivalent simplified mode of ten-story shear frame.

**Figure 5:**
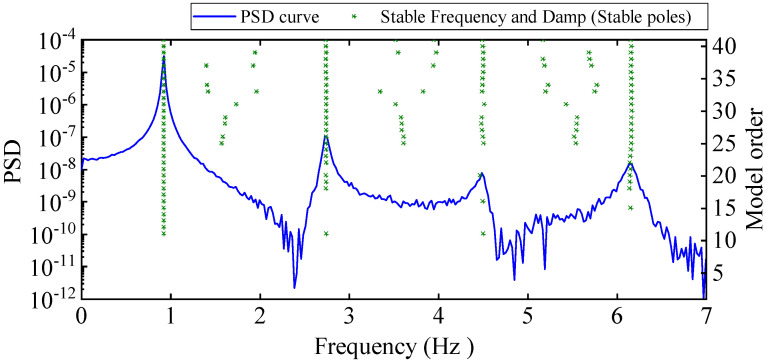
Stability diagram of frame (based on displacement response $x_{8}$ with 5% noise).

A ten-story shear frame structure was established for numerical verification. As shown in Figure 4, it is simplified into a finite degree of freedom system with 10 concentrated particles. The physical parameters of the structure refer to the values of Reference [41], i.e., the mass $m_{i}$ of each degree of freedom is 100 kg, and the stiffness $k_{i}$ between each degree of freedom is 150 kN/m. Using the Cauchy damping assumption, set the damping ratio to 0.01. An impact load of 1 kN was applied horizontally in the 10th degree of freedom, and the displacement response of the structure with free decay for 120 s was collected at a sampling frequency of 14 Hz. To simulate the effect of noise on actual optical dynamic measurements, 5% Gaussian white noise with a mean 0 was added to the displacement responses for all degrees of freedom. It is worth noting that the HRFDD proposed in the paper requires an acceleration sensor for auxiliary measurement. In essence, HRFDD requires a set of dynamic responses with high accuracy as reference data, not limited to the acceleration response, as long as it is a dynamic response with high accuracy. Therefore, the displacement response of one of the degrees of freedom is selected as the reference data of the HRFDD to verify the clustering algorithm. The stability diagram based on the displacement response $x_{8}$ with 5% noise is shown in Figure 5.

**Figure 6:**
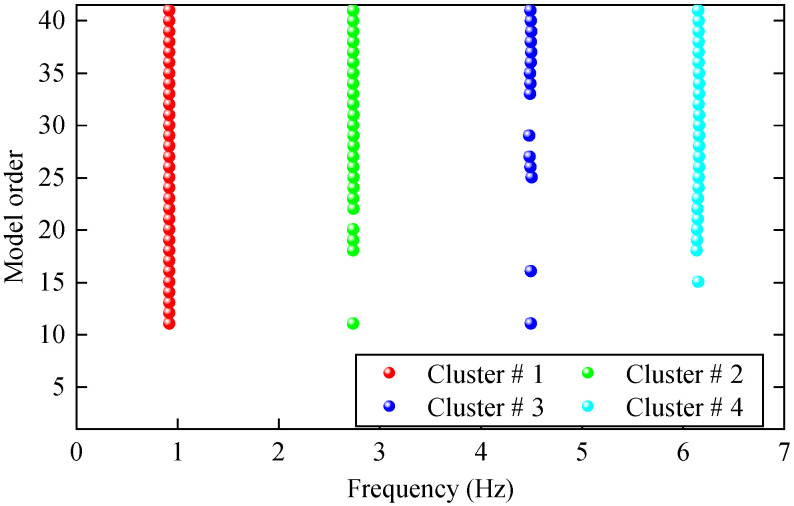
The automated clustering results of frame true poles.

**Figure 7:**
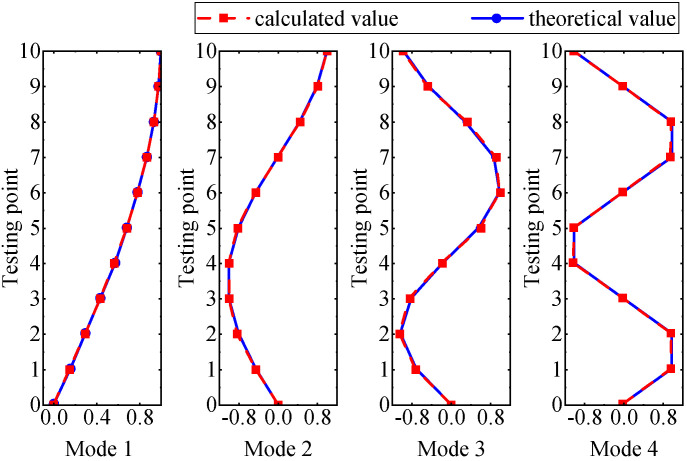
Calculated value and theoretical value of mode shapes.

It can be seen from the graph that there are a large number of scattered stable points at non-natural frequencies. The method proposed in this paper is applied to deal with these scattered stable points for automatic pole identification. In terms of parameter setting, the parameter *Eps* is set to 0.01 according to Equation (14), and the parameter MinPts is set according to Equation (15). In the LSCF calculation process, the system has the minimum model order $p_{min}=2$ and the system has the maximum model order $p_{max}=2$, so *MinPts* = 4. Figure 6 illustrates the automatic clustering results of the true poles of the ten-story shear frame. It can be seen from the figure that after eliminating the interference of false stable points, the stable points retained in each cluster are arranged into a vertical line, and these retained stable points are true poles of the system. Table 1 shows the theoretical values and representative values of the first four order modal parameters of the frame structure, and Figure 7 illustrates the first four mode shapes obtained by the HRFDD method. The modal parameters obtained by the HRFDD method are consistent with the theoretical results. From the perspective of numerical simulation, it is proved that the proposed method for the automatic selection of true poles is effective.

## 4. Modal Analysis of Rectangular Aluminum Plates

### 4.1. Experimental Setup and Tests

**Figure 8:**
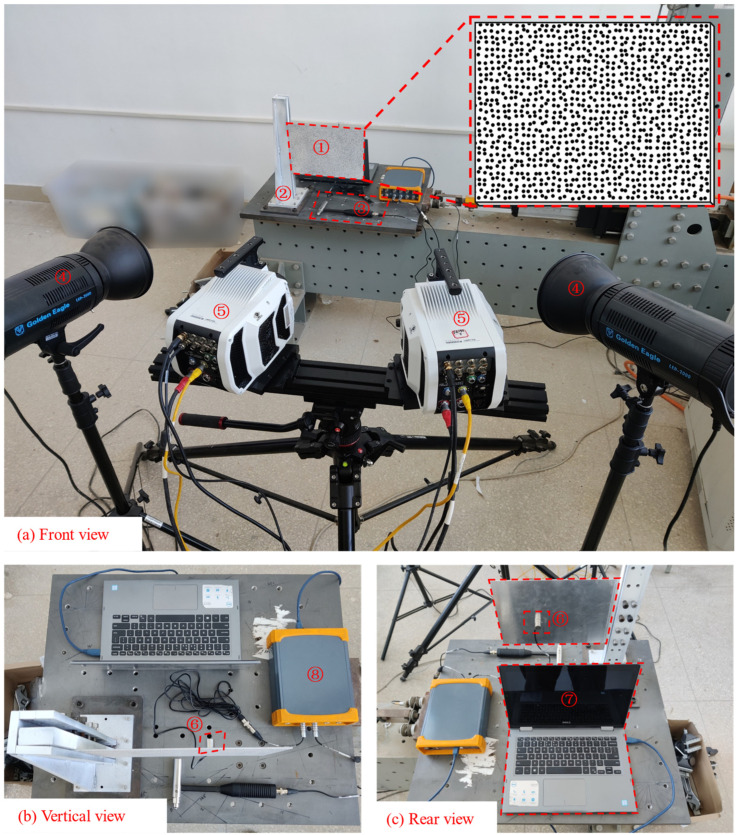
The HRFDD vibration testing system (① aluminum plate, ② customized fixture, ③ impact hammer, ④ lighting, ⑤ high-speed cameras, ⑥ acceleration sensor 1A111E, ⑦ laptop, ⑧ signal analyzer DH5928).

Modal experiments of the rectangular aluminum plate were carried out. The size parameter of the aluminum plate was 320 × 200 × 1.5 mm, the left end of the aluminum plate was clamped by a customized fixture, and the length of the clamped section was 20 mm so that the final effective size of the aluminum plate was 300 × 200 × 1.5 mm. The aluminum plate surface was first sprayed with matte white paint, and then a high-quality, high-contrast black-and-white speckle pattern designed and generated by the “light painting” software [42] was water-transfer printed on it. The experimental system is shown in Figure 8.

In terms of the camera shooting parameter setting of the DIC system, the sampling frame rate was 1000 fps, and the image resolution was set to a full-frame resolution (1280 × 800). Other parameters such as lens aperture, sensitivity, exposure time, and the intensity of photographic light were adjusted simultaneously to achieve the clearest image and the highest black-and-white contrast. In addition, the object distance was adjusted according to the size of the speckle particles in the image, so that the speckle size was exactly four pixels [43,44]. In terms of excitation, an impact load was applied to the back of the aluminum plate along the thickness direction by the impact hammer LC02, causing the cantilever aluminum plate to vibrate freely. The force signal of the impact hammer was collected by the signal analyzer DH5928, and the sampling frequency was 1000 Hz, which was same as the cameras. In terms of dynamic response measurement, a high-frequency optical dynamic measurement system and a 1A111E acceleration sensor were used to simultaneously measure vibration response along the thickness direction (off-plane direction) of the aluminum plate, and the sampling frequency was 1000 Hz. The acceleration sensor acquisition time lasts until the aluminum plate is stationary, that is, until the amplitude of the plate is 0. It is worth noting that due to the limitations of the camera and workstation computer memory, under the above sampling frame number and resolution conditions, the high-speed cameras can only capture 6455 speckle images, that is, the signal acquisition time of the camera is only 6.455 s, while the acceleration sensor acquisition time is much longer than that of the camera, which can easily acquire the acceleration response signal of the entire free attenuation vibration process of the cantilever aluminum plate. Therefore, the accuracy and efficiency of modal analysis supported by optical dynamic measurement will be greatly improved by using the complete acceleration signal as a reference.

**Figure 9:**
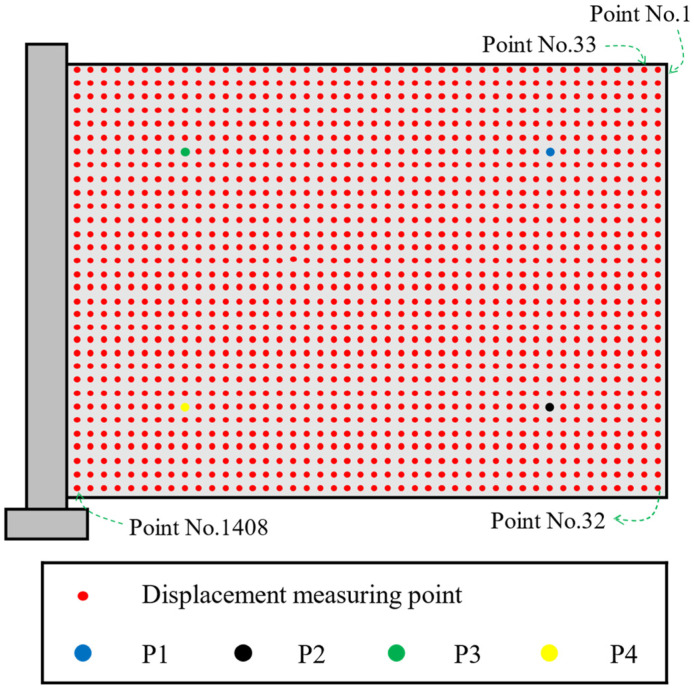
Layout of displacement measuring points on the aluminum plate.

For displacement measurement point arrangement on the aluminum plate surface, 44 measurement points were arranged along the length direction of the aluminum plate, and 32 measurement points were arranged along the width direction, so a total of 1408 displacement measurement points were arranged. It can effectively achieve high-resolution vibration measurement. The detailed arrangement diagram of measurement points is shown in Figure 9. After obtaining speckle image sequences of vibration response, the displacement responses of 1408 measuring points on aluminum plate surface in out-of-plane direction were obtained from these image sequences using Correli^STC^ software (Version 2.1.0.19).

**Figure 10:**
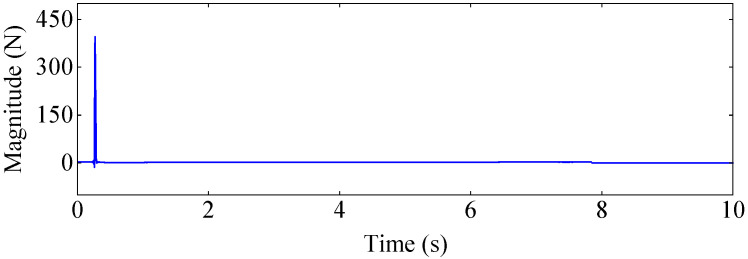
The magnitude of the excitation force.

**Figure 11:**
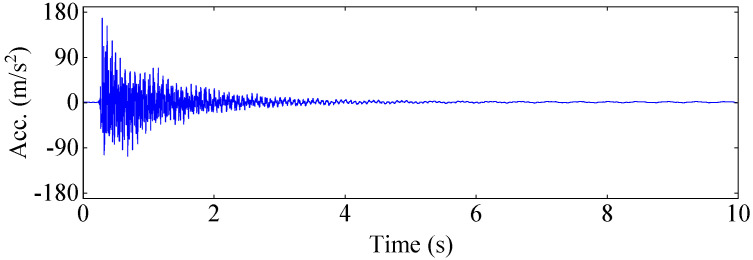
Acceleration response of the aluminum plate.

**Figure 12:**
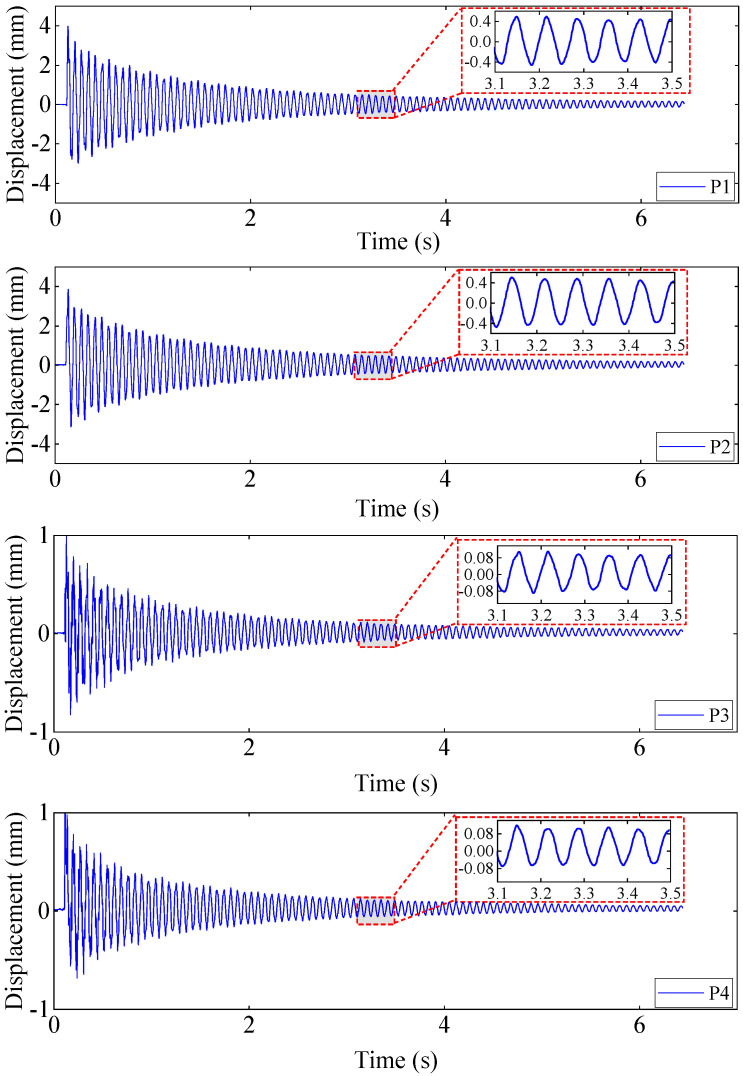
The time–displacement response in the Z direction at four testing points on the surface of the plate.

Figure 10 and Figure 11 show the force and acceleration response signals collected by the signal analyzer DH5928, respectively. Figure 12 illustrates the displacement response signals collected by the high-speed camera at four sample measuring points on the aluminum plate. In order to reduce the random error of experimental measurement, a wavelet threshold de-noising method with double threshold function ${\hat{\omega }}_{j}$ constructed in the study [45] was used to preprocess measurement data.

(16) $${\hat{\omega }}_{j}=\left\{\begin{array}{cc} \mathrm{sgn}\left(\omega_{j}\right)\left(\left|\omega_{j}\right|-\lambda +\frac{\alpha \lambda }{e^{\left(1-\alpha \right)}}\right) & \omega_{j}\geq \lambda \\ \mathrm{sgn}\left(\omega_{j}\right)\frac{\alpha \left(\left|\omega_{j}\right|-\beta \lambda \right)}{\left(1-\beta \right)e^{\left(1-\alpha \right)}} & \lambda \geq \omega_{j}\geq \beta \lambda \\ 0 & other \end{array}\right.$$

where 0<α<1, 0<β<1, and α and β are threshold values and deviation adjustment coefficients, respectively; the threshold function expressed as Equation (16) also performs shrinkage processing on wavelet coefficients in interval [βλ,λ], which makes the reconstructed signal smoother and retains as much local feature information of the original signal as possible.

In the following, the FRF is constructed from the measured force and acceleration response signals to carry out experimental modal analysis, and then only the signals of the displacement response measured by the high-speed camera measurement system were used for operational modal analysis.

### 4.2. Experimental Modal Analysis

The measurement accuracy of the acceleration sensor is much higher than that of the high-speed camera measurement system. The FRF is constructed with the input force signal and output acceleration response signal for experimental modal analysis to obtain an accurate natural frequency and damping ratio of the structure. To weaken the influence of noise and other uncertain factors on the calculation of the FRF, the H_1_ estimation method is used to calculate the frequency response function under single-point excitation [46]. The calculation formula for H_1_ estimation is as follows:

(17) $$H_{1}\left(\omega \right)=\frac{G_{\mathrm{XF}}\left(\omega \right)}{G_{\mathrm{FF}}\left(\omega \right)}=\frac{\frac{1}{N}\sum_{i=1}^{N}X_{i}\left(\omega \right)F_{i}^{*}\left(\omega \right)}{\frac{1}{N}\sum_{i=1}^{N}F_{i}\left(\omega \right)F_{i}^{*}\left(\omega \right)}$$

where $G_{\mathrm{XF}}\left(\omega \right)$ represents the cross-PSD function of the output response and the input excitation, $G_{\mathrm{FF}}\left(\omega \right)$ represents the self-PSD function of the input excitation, $X\left(\omega \right)$ represents the Fourier transform of the output signal, $F\left(\omega \right)$ represents the Fourier transform of the input excitation, ${\left(•\right)}^{*}$ represents the conjugate operation, and N is the total number of times signals were collected during the modal experiment.

After constructing the FRF, the system poles were acquired from the FRF by the LSCF method. It should be noted that in order to avoid mode omission, the model order is often set too large, which directly leads that there are some false poles in the acquired system poles when applying the LSCF method. It is crucial to get rid of the false poles for the accurate identification of modal parameters. Therefore, to effectively distinguish true poles from false poles, the stability diagram is drawn with the frequency as abscissa and the model order as ordinate. When the system pole satisfies Equation (18), it is considered that the pole is stable:

(18) $$\begin{array}{c} \left|\frac{\omega_{p+1}-\omega_{p}}{\omega_{p}}\right|<3\%\\ \left|\frac{\xi_{p+1}-\xi_{p}}{\xi_{p}}\right|<8\% \end{array}$$

**Figure 13:**
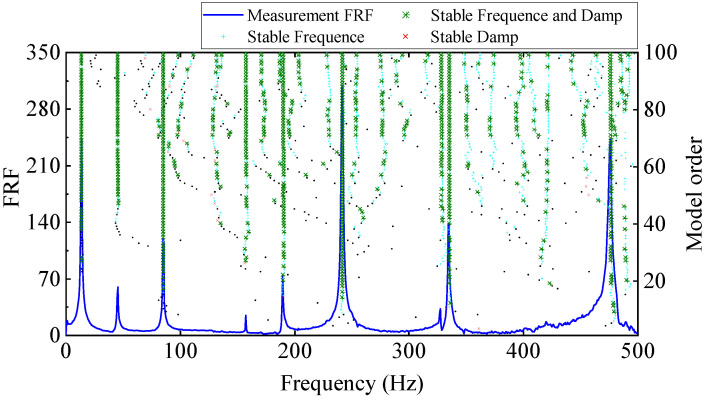
Stability diagram (based on input excitation and output acceleration response).

The stability diagram of experimental modal analysis is shown in Figure 13. In the stability diagram, the true poles are often arranged as a vertical line, while the false poles are scattered. It can be seen from Figure 13 that there are nine modes in the frequency band of 0~500 Hz for the cantilever aluminum plate, among which the 7th and 8th order modes are close-spaced modes. The natural frequencies and damping ratios of the first nine order modes calculated are shown in Table 2.

It is unable to identify the mode shapes of the plate for the test using only one acceleration sensor and fixed single-point excitation. In order to obtain the mode shapes of the plate as the reference for comparisons, finite element analysis (FEA) was performed using ANSYS. The material parameters of the numerical model are set as follows: the elastic modulus is 68 GPa, density is 2760 kg/m^3^, and the Poisson ratio is 0.33; in terms of element type, the four-node quadrilateral element is selected to meshing, each node considered six degrees of freedom, and the boundary conditions are the same as those of the actual experiment, that is, all degrees of freedom of the fixed end nodes are constrained. For the modal analysis algorithm, the Lanczos algorithm is adopted for modal analysis, and the solution results are shown in Table 2. Compared with the frequency results based on LSCF, it can be found that there is little difference in other frequencies except for the fifth natural frequency. It is difficult to achieve fully fixed constraints in practical engineering, so that the inevitable difference in stiffness is the main reason for the difference between the simulation results and the test results. The experimental modal analysis results based on LSCF can be considered accurate when the overall difference is not large. The natural frequencies, damping ratios, and mode shapes obtained based on LSCF and FEA are used as the relatively accurate modal parameter reference data of the cantilever aluminum plate to compare and analyze the OMA results based on high-speed camera measurement data.

### 4.3. Operational Modal Identification Based on Optical Dynamic Measurement

#### 4.3.1. PP Method

The PP method only needs one column of the output response PSD function matrix $G_{\mathrm{yy}}\left(\omega \right)$ to identify the natural frequency, damping ratio, and mode shape. Considering the high signal-to-noise ratio of the displacement response with a large amplitude, the first column of the matrix $G_{\mathrm{yy}}\left(\omega \right)$ is selected for analysis. $G_{m,1}$ is defined as the PSD function of displacement response at measurement point *m* and displacement response at measurement point 1. Getting the first column of $G_{\mathrm{yy}}\left(\omega \right)$ requires computing 1408 PSD functions, so *m* = 1, 2, 3, …, 1408. When calculating the $G_{1,1}$, the windowing in the time domain, overlapping in segments, and averaging in the frequency domain, are not performed, and the number of Fourier transform spectral lines is 2048.

**Figure 14:**
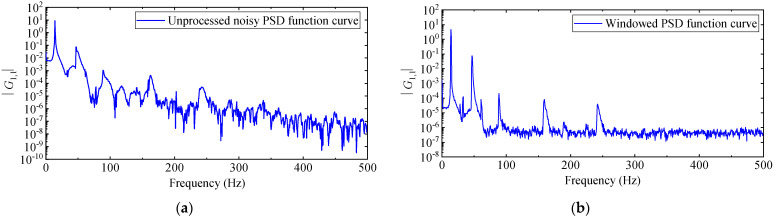
PSD function curve of PP: (**a**) unprocessed noisy PSD function curve; (**b**) windowed PSD curve.

The amplitude diagram of $G_{1,1}$ is shown in Figure 14a. It can be seen from Figure 14a that the effect of peak value identification is very poor; there are many miscellaneous peaks at the low frequency mode, the identification accuracy of natural frequency is not high, and the high frequency mode is basically submerged by noise. The reasons are as follows: On the one hand, due to the limitation of storage capacity, the high-speed camera cannot measure the displacement response of the cantilever aluminum plate in the complete vibration process (from the beginning of vibration to the attenuation of the amplitude to zero), and can only intercept part of the signal (as Figure 12 illustrates). The truncated signal does not attenuate to zero, leading to spectrum energy leakage. The energy at the main peak leaks to the sidelobes, resulting in false peaks appearing on the power spectrum amplitude curve. On the other hand, only performing wavelet threshold denoising can weaken the influence of noise to a certain extent, and the noise of measurement caused by the equipment and algorithm will still cover the peak value of the high frequency mode.

In order to suppress energy leakage, this paper introduces a power–exponential window proposed in reference [47], which is suitable for free attenuation response signals to window the signal in the time domain and attenuate both ends of the signal. To further weaken the influence of noise, the response signal is processed by overlap segmentation and frequency domain averaging. After a lot of tests, the PSD curve has the highest quality when the window size is 2048, the overlap ratio is 50%, and the number of Fourier transform lines is 2048. The $G_{1,1}$ amplitude curve after data processing is shown in Figure 14b, with clearer peaks and fewer “spectral spurs”, which is more conducive to observing the modal distribution and picking up peak frequencies.

**Figure 15:**
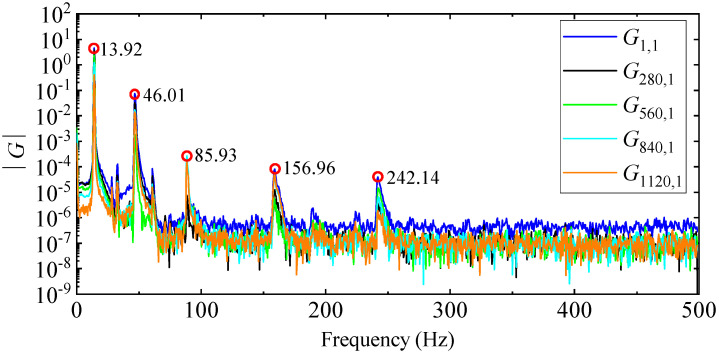
Amplitude curves of several PSD functions.

However, since all modes cannot be accurately picked up by the $G_{1,1}$ amplitude curve alone, peaks are picked up from multiple PSD amplitude curves to avoid missing modes, as shown in Figure 15. The damping ratio is calculated by the half-power bandwidth method after the resonance peak position is determined, and the mode shape is obtained according to the real part of the PSD function at the peak position. It should be noted that, in terms of computational efficiency, the PP method takes 8 s, of which 3 s are spent calculating the 1408 PSD functions in MATLAB, and 5 s are spent manually picking up peaks from the PSD amplitude curves and calculating damping ratios and mode shapes.

#### 4.3.2. EFDD Method

**Figure 16:**
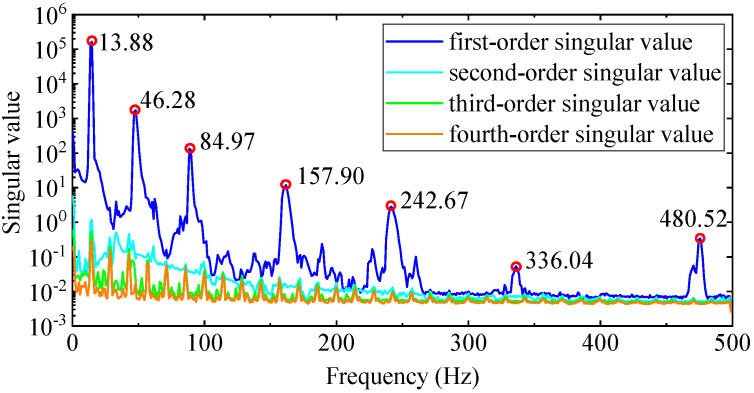
The first four singular value spectrum curves.

Using the EFDD method requires constructing the PSD function matrix $G_{\mathrm{yy}}\left(\omega \right)$ of output response first, and it has to calculate 1,982,464 PSD functions in total. The input parameters calculated by the PP method are still used to calculate the power spectrum. After the calculation is completed, the SVD is performed on matrix $G_{\mathrm{yy}}\left(\omega \right)$ of each frequency point ω, and the SVD calculation results of all discrete frequency points are retained. The singular value spectra are drawn according to the singular values of each discrete frequency point. The four order singular value spectrum curves of the aluminum plate are shown in Figure 16, from which seven order modes with high reliability can be identified. The first column singular vector of the left singular matrix at the peak frequency is normalized to acquire the mode shape, and the damping ratio is calculated by Equations (5) and (6). In terms of computational efficiency, the EFDD method takes more than half an hour. The time is mainly spent on calculating the PSD function matrix $G_{\mathrm{yy}}\left(\omega \right)$ of the output response, and secondly, on calculating the SVD of the matrix $G_{\mathrm{yy}}\left(\omega \right)$ at discrete frequency points.

#### 4.3.3. LSCF Method

**Figure 17:**
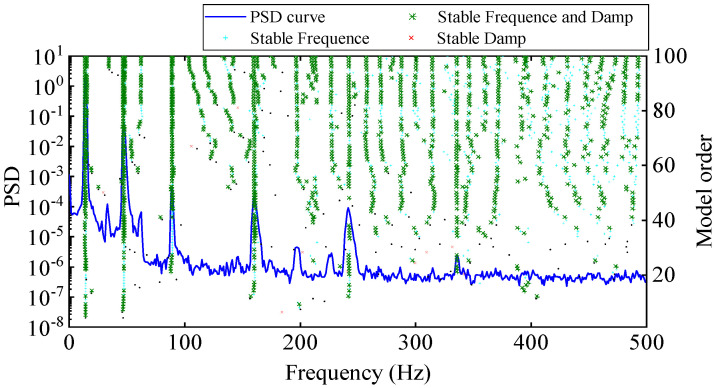
Stability diagram (based on displacement data measured by high-speed camera).

Similar to the EFDD method, using the LSCF method also first needs constructing of the PSD function matrix $G_{\mathrm{yy}}\left(\omega \right)$. The PSD function is fitted to a right matrix fractional model (RMFM) based on the least-squares fitting method, and then the companion matrix is established according to the denominator coefficient, and the system poles are obtained by the SVD of the companion matrix. Figure 12 is the stability diagram constructed using only output responses measured by the DIC, where the threshold criteria for stable poles are consistent with Equation (18). According to the distribution of stable points, the six order system poles with high reliability are selected from Figure 17, and the mode shapes are solved according to the least-squares frequency domain method. It is worth noting that in terms of computational efficiency, the LSCF method takes more than half an hour, with these three processes taking the longest time: constructing the PSD function matrix of the output response, parametric least-squares fitting, and mode shape least-squares evaluation.

### 4.4. The Improved Operating Modal Analysis Approach

**Figure 18:**
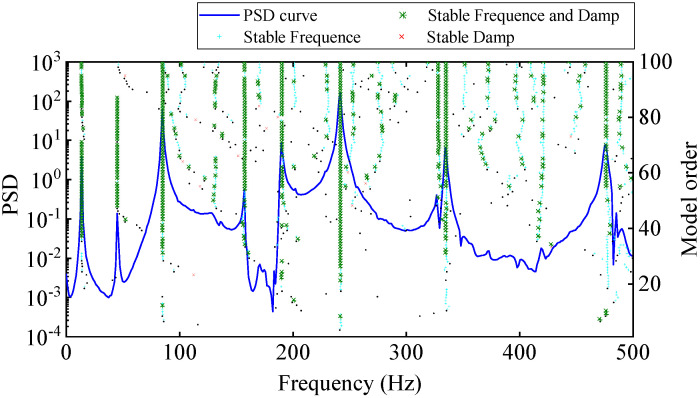
Stability diagram of the plate (based on a set of acceleration response data).

**Figure 19:**
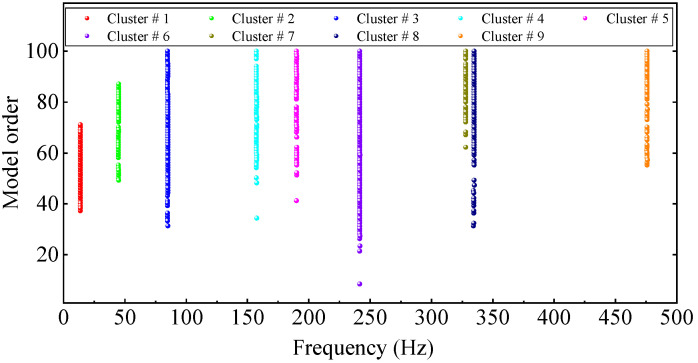
True pole automated clustering results of the rectangular plate.

To use the HRFDD method to identify the modal parameters of the cantilever aluminum plate, firstly, the LSCF method is applied to acquire the natural frequency and damping ratio from a set of response data obtained by the acceleration sensor. The stability diagram constructed using only acceleration data is shown in Figure 18, where the threshold criteria for the stable poles are consistent with Equation (18). The automatic clustering results of the true poles are illustrated in Figure 19. It can be clearly seen from Figure 19 that there are nine modes in the range of 0~500 Hz, and the stable points are distributed in vertical lines at each natural frequency. The true poles of each cluster are averaged and the average value is taken as the representative value of the mode of this order, as shown in Table 3. The modal data in Table 3 are basically consistent with Table 2, which proves that the proposed method can accurately and automatically select the true poles.

After obtaining the accurate natural frequency and damping ratio, the PSD matrix at the natural frequency is calculated by the Welch method, and the mode shape can be obtained by the SVD decomposition of the PSD matrix. In terms of calculation time, the HRFDD method only needs to calculate the PSD matrix at the natural frequency, so the calculation amount is very small. It takes only 18 s to identify the first nine order modal parameters of the plate.

### 4.5. Comparison and Analysis of Results

**Figure 20:**
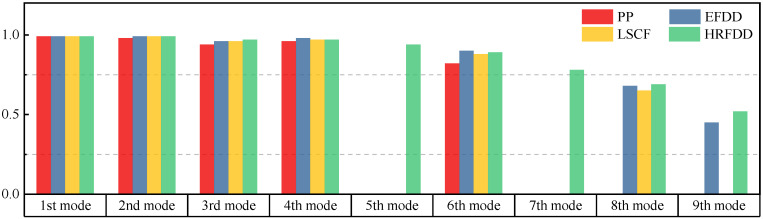
Comparison of MAC values obtained by comparing mode shapes obtained from FEA with those obtained from operational modal analysis (PP, EFDD, LSCF, and the proposed HRFDD).

The comparison results of natural frequencies and damping ratios of the above methods are shown in Table 4, where the reference values are modal data identified by experimental modal analysis. Table 5 shows the identification results of mode shapes, and “/” in the table indicates modes that cannot be identified by the corresponding method. In order to judge the accuracy of the mode shape identified by the above methods, the MAC is used to evaluate them. The MAC value of the above methods, which is calculated based on the measured mode shapes obtained by each modal analysis method and the corresponding mode shapes obtained by FEA, is illustrated in Figure 20. The closer the MAC value is to 1, the higher the modal correlation is, indicating that the measured mode shape is closer to the numerical simulation reference result, and the modal shape of FEA is normalized during the calculation.

Compared with other identification methods, the HRFDD method can accurately identify all the modes of the cantilever aluminum plate without missing modes. It can be seen from Figure 20 that the MAC value of HRFDD is higher than that of the PP method, and there is little difference between the HRFDD method with the EFDD method and LSCF method, which indicates that the HRFDD method inherits the noise reduction characteristics of SVD and can identify mode shapes with high resolution. In addition, the HRFDD method also excels in terms of calculation time. Compared with the EFDD and LSCF method, which take more than half an hour of calculation, the HRFDD only takes 18 s. Although the PP method takes 8 s, which is less than the HRFDD, the PP method is far less accurate than the HRFDD. In summary, the HRFDD method can minimize the huge calculation amount on the premise of ensuring identification accuracy. The calculation efficiency is extremely high, basically meeting the requirements of online evaluation for structural health monitoring.

## 5. Conclusions

In this paper, an automated high-resolution OMA is proposed, which adopts optical dynamic measurement assisted by an additional acceleration sensor to acquire structural vibration responses; the suitable clustering algorithm is developed to identify the modal parameters automatically. The vibration test and numerical simulation are carried out to verify the method, and the following conclusions are obtained:

1. The experiments demonstrate that the classical OMA frequency domain method cannot accurately and efficiently identify all modes of the cantilever aluminum plate in the frequency range of 0~500 Hz from optical measurement data with low signal-to-noise ratios and short acquisition times;
2. The HRFDD method proposed in this paper has a higher accuracy and completeness of modal identification, and can identify all modes of the plate in the range of 0~500 Hz with high accuracy and no missing;
3. The experiments show that the HRFDD method greatly improves computational efficiency compared with the classical OMA frequency domain method, essentially meeting the requirements for real-time online monitoring. It can be applied to structural health monitoring;
4. Numerical simulation and laboratory experiments indicate that the real poles automatic selection method constructed by integrating the DBSCAN and the three sigma criterion has further enhanced the usage efficiency of the HRFDD.

The optical dynamic measurement method can obtain a high-resolution displacement response, which is an excellent tool for structural dynamic response measurement. When applied to structural health monitoring, optical dynamic measurement faces two major problems: noise influence and computational efficiency, which need to be improved. The classical operational modal analysis based on optical dynamic measurement has a poor ability to obtain a high-resolution result as the experiment shows. The HRFDD proposed in this paper effectively reduces the noise influence on measurement and improves computational efficiency.

There are numerous self-made script functions to be called in the automatic modal parameter identification process so that the software of automatic modal parameter identification suitable for high-frequency optical dynamic measurement will be developed in the subsequent research to simplify the cumbersome steps and facilitate the user’s operation. The proposed real pole selection and HRFDD method will be integrated into the software to facilitate the subsequent research on structural damage identification based on modal parameters. In light of the above studies, future research will focus on thin-walled structural damage detection based on modal parameters, especially damage localization based on high-resolution mode shapes.

## Figures and Tables

**Table 1:** Theoretical value (TV) and modal representative values (RVs) of natural frequency f and damping ratio ξ.

Mode Order	1st Mode		2nd Mode		3rd Mode		4th Mode	
	TV	RV	TV	RV	TV	RV	TV	RV
f (Hz)	0.9213	0.9213	2.7433	2.7431	4.5040	4.5010	6.1640	6.1643
ξ (%)	1	1.0021	1	1.0191	1	1.0536	1	1.0130

**Table 2:** Experimental modal identification using accelerometers and FEA.

Mode Order	Natural Frequency (Hz)		Damping Ratio (%)	Mode Shape	Mode Order	Natural Frequency (Hz)		Damping Ratio (%)	Mode Shape
	LSCF	FEA	LSCF	FEA		LSCF	FEA	LSCF	FEA
1st	13.56	13.71	0.35	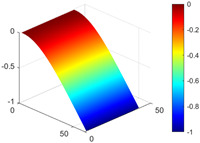	6th	242.02	245.57	0.43	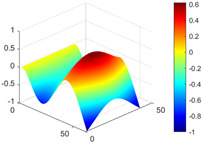
2nd	45.40	45.64	0.23	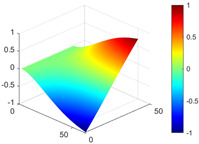	7th	328.71	316.59	0.46	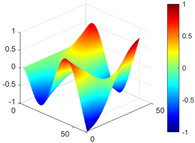
3rd	85.12	85.07	0.77	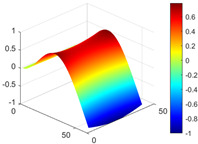	8th	335.57	337.06	0.30	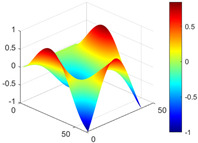
4th	157.63	154.53	0.37	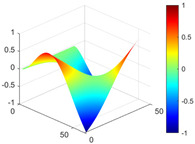	9th	476.44	476.22	0.43	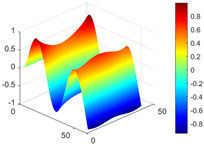
5th	190.25	211.79	0.55	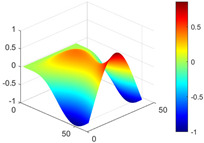					

**Table 3:** Representative values of the first natural frequency f and damping ratio ξ of the rectangular plate.

Mode Order	1st	2nd	3rd	4th	5th	6th	7th	8th	9th
f (Hz)	13.58	45.41	85.16	157.69	190.25	242.12	328.99	335.63	476.68
ξ (%)	0.34	0.24	0.77	0.37	0.54	0.46	0.42	0.32	0.46

**Table 4:** Comparison of natural frequencies and damping ratios measured using the accelerometer(reference), the proposed HRFDD, and three operating modal identification methods (PP, EFDD, and LSCF) based on displacement data measured by a high-speed camera.

Mode Order	Natural Frequency Damping Ratio	Reference	HRFDD	PP	EFDD	LSCF
1st mode	Frequency (Hz)	13.56	13.58	13.92	13.88	13.67
	Damping ratio (%)	0.35	0.35	0.47	0.42	0.34
2nd mode	Frequency (Hz)	45.40	45.40	46.01	46.28	45.99
	Damping ratio (%)	0.23	0.24	0.53	0.30	0.25
3rd mode	Frequency (Hz)	85.12	85.14	85.93	84.97	85.64
	Damping ratio (%)	0.77	0.78	0.69	0.74	0.68
4th mode	Frequency (Hz)	157.63	157.69	156.96	157.90	158.02
	Damping ratio (%)	0.37	0.37	0.74	0.28	0.32
5th mode	Frequency (Hz)	190.25	190.24	/	/	/
	Damping ratio (%)	0.55	0.56	/	/	/
6th mode	Frequency (Hz)	242.02	242.09	242.14	242.67	242.81
	Damping ratio (%)	0.43	0.45	0.48	0.36	0.52
7th mode	Frequency (Hz)	328.71	328.96	/	/	/
	Damping ratio (%)	0.46	0.42	/	/	/
8th mode	Frequency (Hz)	335.57	335.61	/	336.04	336.03
	Damping ratio (%)	0.30	0.32	/	0.45	0.39
9th mode	Frequency (Hz)	476.44	476.67	/	480.52	/
	Damping ratio (%)	0.43	0.46	/	0.37	/

**Table 5:** Mode shapes obtained by the proposed HRFDD and three operating modal identification methods (PP, EFDD, and LSCF) based on displacement data measured by a high-speed camera.

Mode Order	HRFDD	PP	EFDD	LSCF
1st	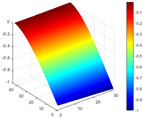	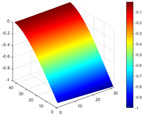	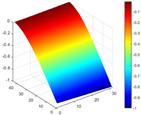	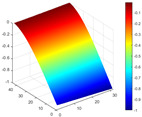
2nd	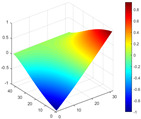	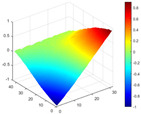	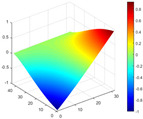	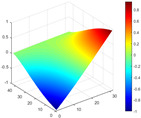
3rd	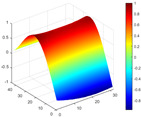	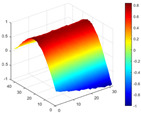	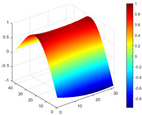	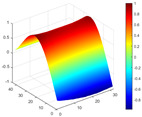
4th	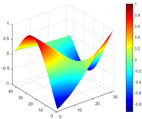	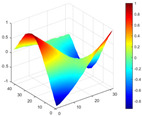	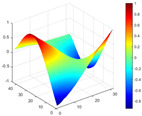	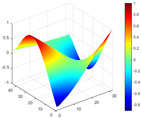
5th	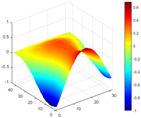	/	/	/
6th	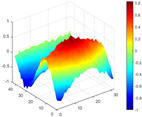	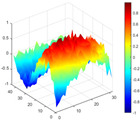	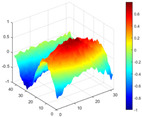	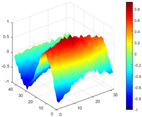
7th	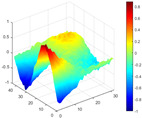	/	/	/
8th	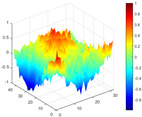	/	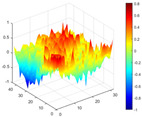	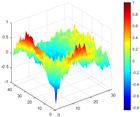
9th	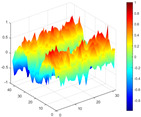	/	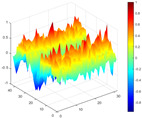	/

## Data Availability

The original contributions presented in the study are included in the article, further inquiries can be directed to the corresponding author.
